# Supplementary material for: White matter tracts associated with iTBS-induced heart rate deceleration and treatment response in major depressive disorder
Source: Transl Psychiatry. 2025 Oct 20;15:424. doi: 10.1038/s41398-025-03646-3 (PMC12537985; doi:10.1038/s41398-025-03646-3)
Supplement: Supplementary file 1 — Supplementary Legends [file 41398_2025_3646_MOESM1_ESM.docx]

**Supplementary Figure 1: Previously reported clinical findings.** a) Montgomery–Åsberg Depression Rating Scale (MADRS) mean total scores in the sham-active and active-sham groups from V1 to V6. b)

Pearson correlation between relative delta MADRS and the corresponding slope in RR intervals after

45s of stimulation on the first day of the week. Slope value represents the median of the sessions. c)

Comparison of median slope values after 45s of stimulation. Paired t-test t(79) = 2.54, p = 0.0132.

**Supplementary Figure 2: Correlational tractography.** Varying t-score thresholds were inspected to

select local connectomes (T: 1, 2 and 3) at different significance levels (p FDR < 0.05, < 0.1 and < 0.2)

as suggested by the DSI-Studio documentation (<https://dsi-studio.labsolver.org/doc/gui_cx.html>). The

black frame indicates the range of values selected for further analysis in this work.

**Supplementary Table 1: Baseline scans.** Results from correlational tractography based on the HCP-

1065 tractography atlas

**Supplementary Table 2: Delta scans.** Results from correlational tractography based on the HCP-1065

tractography atlas
