# Supplementary figures and images for: White matter tracts associated with iTBS-induced heart rate deceleration and treatment response in major depressive disorder
Source: Transl Psychiatry. 2025 Oct 20;15:424. doi: 10.1038/s41398-025-03646-3 (PMC12537985; doi:10.1038/s41398-025-03646-3)

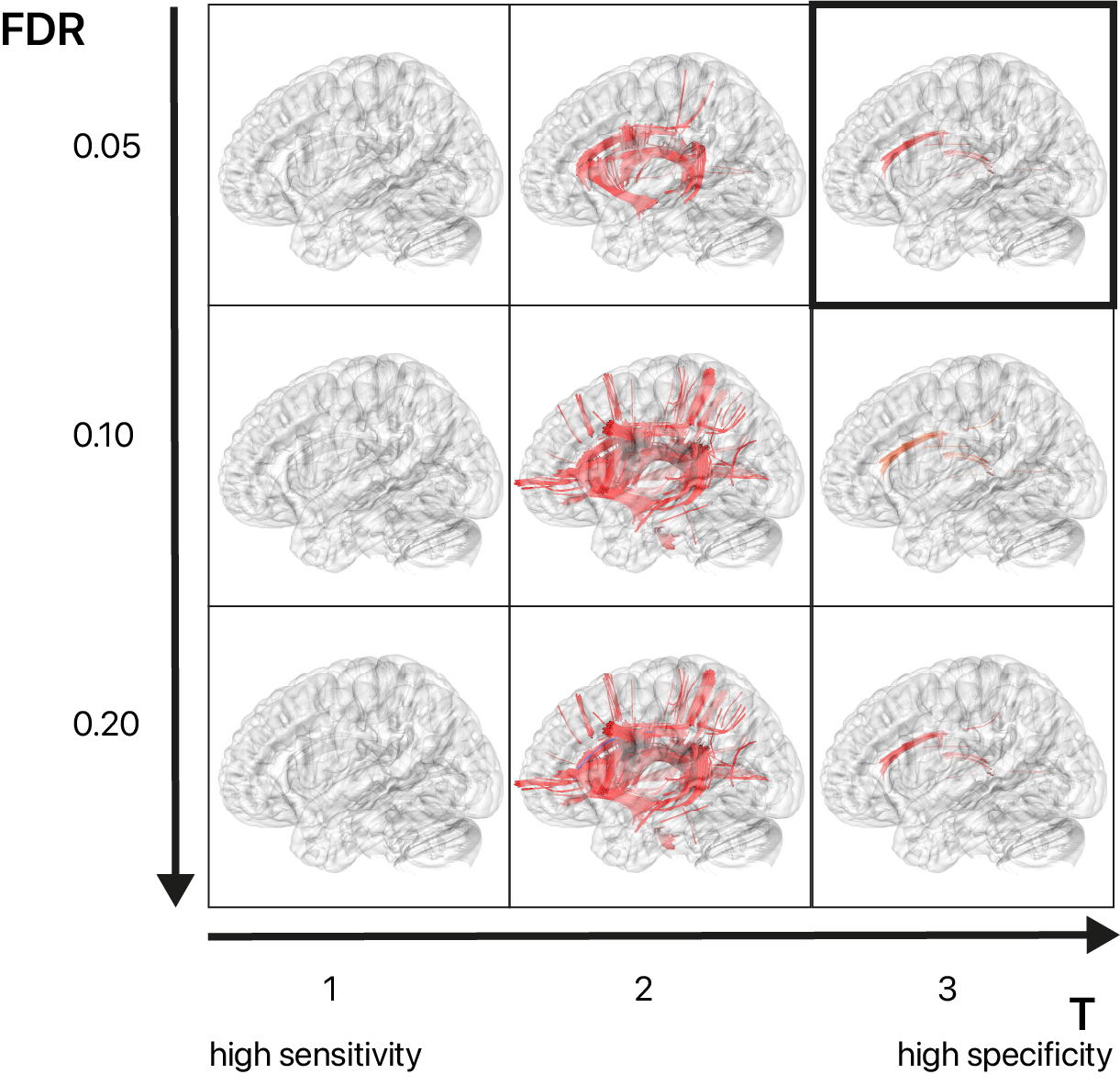

Supplement: Supplementary file 2 — Supplementary Figure 1 [file 41398_2025_3646_MOESM2_ESM.png]

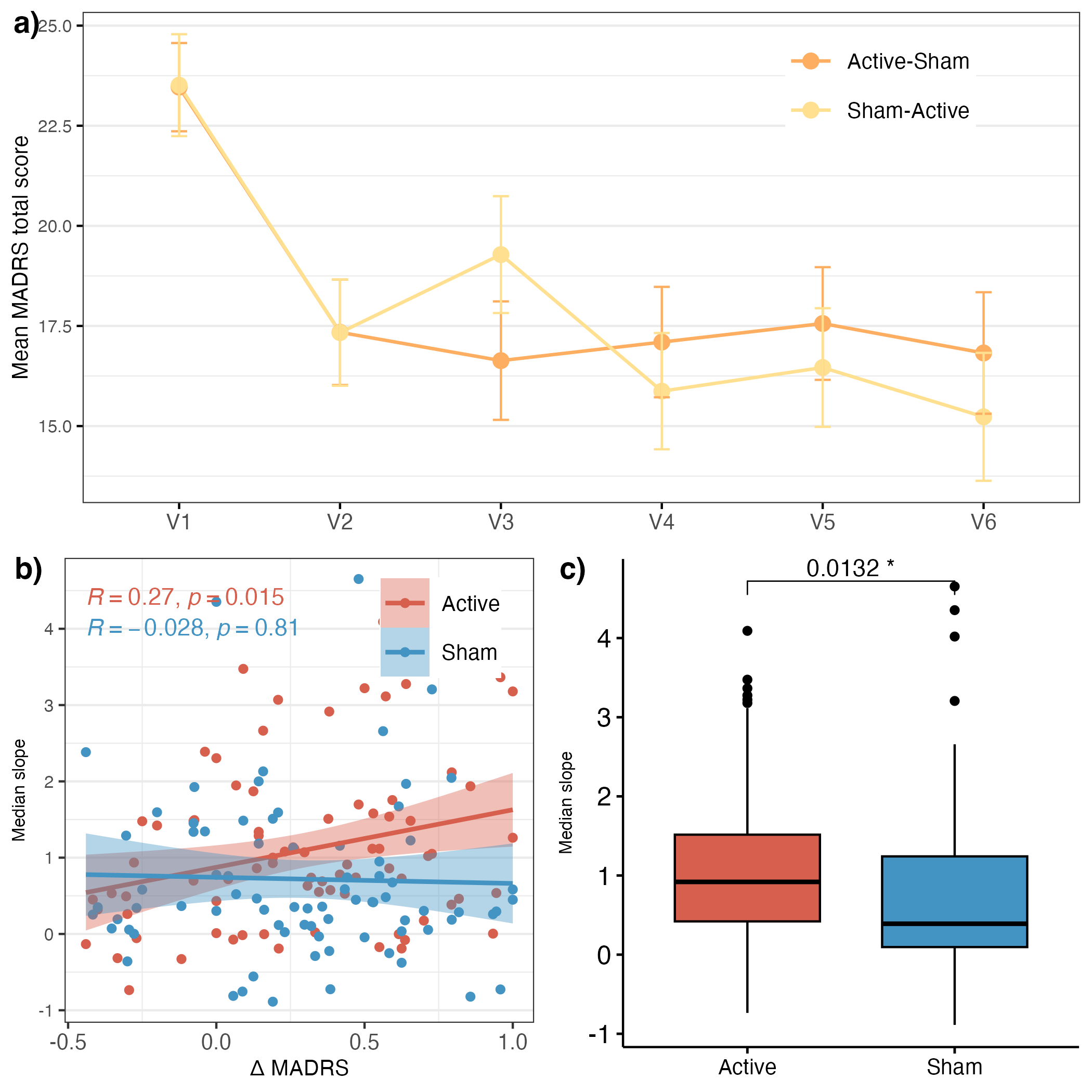

Supplement: Supplementary file 3 — Supplementary Figure 2 [file 41398_2025_3646_MOESM3_ESM.png]
