# Supplementary material for: White matter tracts associated with iTBS-induced heart rate deceleration and treatment response in major depressive disorder
Source: Transl Psychiatry. 2025 Oct 20;15:424. doi: 10.1038/s41398-025-03646-3 (PMC12537985; doi:10.1038/s41398-025-03646-3)
Supplement: Supplementary file 4 — Supplementary Table 1: [file 41398_2025_3646_MOESM4_ESM.docx]

| Baseline scans deltaMADRS | | |
| --- | --- | --- |
| FA_T_ deltaMADRS | Positive (132) | 68.1818% Cingulum_Parolfactory_R  11.3636% Fornix_R  10.6061% Cingulum_Frontal_Parietal_R  3.78788% Inferior_Fronto_Occipital_Fasciculus_R  1.51515% Corpus_Callosum_Body  0.757576% Corpus_Callosum_Forceps_Major |
|  | Negative (0) |  |
| MD_T_ deltaMADRS | Positive (0) |  |
|  | Negative (2077) | 55.4165% Fornix_R  22.7732% Fornix_L  18.1993% Thalamic_Radiation_Superior_R  2.02215% Thalamic_Radiation_Posterior_R  0.818488% Optic_Radiation_R  0.288878% Thalamic_Radiation_Anterior_R  0.240732% Corpus_Callosum_Body  0.144439% Corpus_Callosum_Forceps_Major  0.0481464% Corpus_Callosum_Tapetum |
| RD_T_ deltaMADRS | Positive (0) |  |
|  | Negative (1701) | 52.5573% Fornix_R  23.1041% Fornix_L  20.047% Thalamic_Radiation_Superior_R  2.46914% Thalamic_Radiation_Posterior_R  0.705467% Optic_Radiation_R  0.235156% Thalamic_Radiation_Anterior_R  0.176367% Corpus_Callosum_Body  0.117578% CNIII_R  0.0587889% Dentatorubrothalamic_Tract_R |
| V1 Slope | | |
| FA_T_ Slope | Positive (9) | 77.7778% Corpus_Callosum_Forceps_Major  11.1111% Superior_Longitudinal_Fasciculus1_L |
|  | Negative (991) | 60.444% Fornix_R  15.4389% Fornix_L  11.2008% Cingulum_Frontal_Parietal_R  8.07265% Thalamic_Radiation_Superior_L  1.41271% Cingulum_Parolfactory_R  1.10999% Cingulum_Frontal_Parietal_L  1.00908% Corpus_Callosum_Forceps_Major  0.504541% Cingulum_Parolfactory_L  0.302725% Cingulum_Frontal_Parahippocampal_R  0.100908% Corpus_Callosum_Body |
| MD_T_ Slope | Positive (1649) | 63.8569% Fornix_R  21.5282% Fornix_L  8.91449% Thalamic_Radiation_Superior_L  2.00121% Cingulum_Frontal_Parietal_L  1.94057% Corpus_Callosum_Forceps_Major  0.545785% Thalamic_Radiation_Anterior_L  0.303214% Corpus_Callosum_Body  0.242571% Superior_Longitudinal_Fasciculus1_L  0.121286% Optic_Radiation_R  0.0606428% Thalamic_Radiation_Posterior_R |
|  | Negative (0) |  |
| RD_T_ Slope | Positive (1864) | 66.3627% Fornix_R  18.0258% Fornix_L  9.12017% Thalamic_Radiation_Superior_L  1.93133% Corpus_Callosum_Forceps_Major  0.858369% Thalamic_Radiation_Anterior_L  0.375536% Corpus_Callosum_Body  0.26824% Superior_Longitudinal_Fasciculus1_L  0.160944% Corpus_Callosum_Tapetum  0.107296% Optic_Radiation_R  0.0536481% Optic_Radiation_L |
|  | Negative (0) |  |
