# Supplementary material for: White matter tracts associated with iTBS-induced heart rate deceleration and treatment response in major depressive disorder
Source: Transl Psychiatry. 2025 Oct 20;15:424. doi: 10.1038/s41398-025-03646-3 (PMC12537985; doi:10.1038/s41398-025-03646-3)
Supplement: Supplementary file 5 — Supplementary Table 2: [file 41398_2025_3646_MOESM5_ESM.docx]

| Delta scans deltaMADRS | | |
| --- | --- | --- |
| FA deltaMADRS | Positive (342) | 80.9942% Cingulum_Parolfactory_L  11.1111% Cingulum_Frontal_Parietal_L  4.09357% Superior_Longitudinal_Fasciculus1_L  2.04678% Corpus_Callosum_Forceps_Major  0.584795% Fornix_R  0.292398% Corpus_Callosum_Body |
|  | Negative (35) | 42.8571% Fornix_L  40% Cingulum_Parolfactory_R  14.2857% Cingulum_Frontal_Parietal_R  2.85714% Corpus_Callosum_Tapetum |
| MD deltaMADRS | Positive (105) | 53.3333% Thalamic_Radiation_Superior_R  25.7143% Fornix_L  16.1905% CNIII_R  2.85714% Corpus_Callosum_Forceps_Major  0.952381% Middle_Cerebellar_Peduncle |
|  | Negative (78) | 48.7179% Thalamic_Radiation_Anterior_L  33.3333% Thalamic_Radiation_Superior_L  7.69231% Corpus_Callosum_Forceps_Major  3.84615% Fornix_R  2.5641% Corpus_Callosum_Tapetum  1.28205% Cingulum_Frontal_Parahippocampal_L |
| RD deltaMADRS | Positive (100) | 52% Thalamic_Radiation_Superior_R  24% Fornix_L  18% CNIII_R  2% Reticular_Tract_R  1% Middle_Cerebellar_Peduncle |
|  | Negative (144) | 33.3333% Thalamic_Radiation_Superior_L  26.3889% Cingulum_Parolfactory_L  25% Thalamic_Radiation_Anterior_L  3.47222% Corpus_Callosum_Forceps_Major  2.08333% Fornix_R  1.38889% Reticular_Tract_L  0.694444% Superior_Longitudinal_Fasciculus1_L |
